# Notes and News

**Published:** 1895-06-01

**Authors:** 


### Notes and News.

(Collected and Communicated.)

Dr. Fancourt Barnes has been unanimously elected Consulting Physician to the British Lying-in Hospital.

The foundation-stone of the new Samaritan Hospital for Women, at Glasgow, was laid last week by Lord Blythswood. £5,000 or £6,000 are yet needed to complete the buildings.

The contemplated additions to the Brooklyn Naval Hospital will consist of a wing joining the present building, in which will be included a completely equipped operation theatre. It appears likely that a Hahnemannian hospital for the insane will be established in Pennsylvania, a large number of petitions for its erection having been presented to the Legislature.

The Chelsea Hospital for Women was gaily decorated inside and out on Monday last in anticipation of a Royal visit, the Duchess of Teck having consented to be present at the opening and naming of a new ward. Her Royal Highness was received at the entrance of the building by Sir Algernon Borthwick, M.P., the Chairman of the Council, and other members, and was presented with a bouquet of pink roses on behalf of the Ladies' Committee by the little daughter of Mr. O'Callaghan, the senior surgeon, afterwards going into all the wards and distributing flowers to the patients, with a kindly word to each. The door of the new ward was then formally unlocked, and a tablet bearing the name "Mary Adelaide" affixed to it, while the Duchess declared the ward open, remarking that she had much pleasure in naming it the "Mary Adelaide Ward," 'in memory of a visit paid to the hospital at a time when it was rather under a shadow, but which we all hope is now entirely removed." Afterwards the Princess received purses in aid of the general funds of the hospital, amounting to about £260. The Princess was presented with a vase, in the form of a golden slipper, as a memento of the occasion on leaving the hospital.

Mr. Alexander Hayes, the assistant secretary of St. Mary's Hospital, has been appointed secretary to the Metropolitan Convalescent Institution, vice Mr. Charles Holmes, who retires after more than forty-two years' service.

It is understood that the French Army Medical Service is to be increased. This has been regarded as a most desirable step in many quarters for some time past. The Government are also believed to have agreed that medical students shall only do their military service after they have finished their studies.

The performance of Dion Boucicault's "Arrah-Na-Pogue," by the Marlow Dramatic Club, in aid of the funds of the Great Northern Central Hospital, met with much appreciation from the audience, and no doubt the proceeds will materially benefit the institution.

Several additions have been made recently to hospital buildings. Last week the Earl of Mount-Edgcumbe laid the foundation-stone of a cottage hospital at Liskeard, provided by Mr. Passmore Edwards at an expenditure of £2,000. At Fleetwood a cottage hospital was opened by Colonel Foster. The contributions have reached £2,325, and £250 is required in addition to provide for furnishing. A new wing has been added to the West of Scotland Convalescent Home at Dunoon. The addition will add fifty beds to the institution, and has cost about £5,000.

Amongst many other charitable institutions which have benefited under the will of the late Mr. E. T. Perry, sugar broker, of Liverpool, we notice the following: Royal Infirmary; Convalescent Fund, Northern Hospital; Convalescent Fund, Royal Southern Hospital; Convalescent Fund of the Infirmary for Children, all Liverpool institutions, each £1,000. We are also glad to see that these generous bequests are made "after providing for his widow" and remembering various relations and friends. It not infrequently happens that people who largely benefit public charities forget the simple and all-important fact that "charity begins at home" when disposing of their worldly goods.

At a recent quarterly meeting of the General Committee of the Walsall and District Hospital, the question of the addition of medical wards, at present under consideration by the authorities, was discussed. It will be remembered that, owing to the damage lately done to the building in a severe storm, extensive repairs and rebuilding had to be undertaken, and it became matter for consideration as to whether a necessary increase of beds for medical cases could not be carried out simultaneously. With regard to funds, it was stated by one of the committee that the workpeople readily gave their penny and halfpenny subscriptions, and contributions from that source had much increased, indeed, had been almost trebled in the last two or three years; but the masters were not as willing to co-operate as could be wished. A resolution was finally passed suggesting that a conference of representatives of trade associations, friendly societies, and medical aid societies should be held for further consideration as to the best steps to be taken, and the sum it was necessary to secure, for the addition of the proposed twenty-five medical beds.

